# Cohort Profile: The AGES 2003 Cohort Study in Aichi, Japan

**DOI:** 10.2188/jea.JE20100135

**Published:** 2011-03-05

**Authors:** Akihiro Nishi, Katsunori Kondo, Hiroshi Hirai, Ichiro Kawachi

**Affiliations:** 1 Department of Society, Human Development, and Health, Harvard School of Public Health, Boston, MA, USA; 2 Department of Public Health, Graduate School of Medicine, The University of Tokyo, Tokyo, Japan; 3 Center for Well-being and Society, Nihon Fukushi University, Nagoya, Japan

**Keywords:** Japanese longevity, social support, social capital, cohort profile, the AGES project

## Abstract

**Background:**

The longevity of Japanese is thought to be associated with psychosocial factors such as sense of coherence, social support, and social capital. However, the actual factors responsible and the extent of their contribution to individual health status are not known.

**Methods:**

The Aichi Gerontological Evaluation Study (AGES) 2003 Cohort Study is a prospective cohort study of community-dwelling, activities of daily living-independent people aged 65 or older living in 6 municipalities in Chita peninsula, Aichi Prefecture, Japan. Information on psychosocial factors and other individual- and community-level factors was collected in the second half of 2003 using a baseline questionnaire. Vital status and physical and cognitive decline have been followed using data derived from long-term care insurance certification. Geographical information on the study participants was also obtained.

**Results:**

A total of 13 310 (6508 men; 6802 women) study participants were registered in the study. For an interim report, we followed the cohort for 48 months, yielding 24 753 person-years of observation among men and 26 456 person-years among women.

**Conclusions:**

The AGES 2003 Cohort Study provides useful evidence for research in social epidemiology, gerontology, and health services.

## INTRODUCTION

“Why are Japanese living longer?”^1^ The question of Japanese longevity is fascinating, and the global community wants to know more about this phenomenon. In 2008, life expectancy was 79.19 years in Japanese men and 85.99 years in Japanese women, which outrank those of nearly all developed and developing countries.^2^^,^^3^ While some believe that genetic differences between Japanese and Western populations are a factor, research suggests that this is an unlikely explanation.^4^^,^^5^

Resources in society can improve the general health of individuals.^6^^,^^7^ Many people agree that the health of individuals is affected by several aspects of society, such as cohesion, lifestyle, customs, family structure, culture, and religious beliefs, all of which are generated through each country’s or community’s complexity and context over a period of many years.^6^^,^^8^ Large-scale cohort studies are being conducted in Japan to reveal the reasons for Japanese longevity, but these studies are limited in their ability to make causal inferences because of the complexity of the underlying mechanisms.^9^^–^^20^

In addition to the work of scholars, the national and local governments are also addressing the issue of aging in Japan. For instance, the nationwide universal long-term care insurance system was started in 2000,^21^^,^^22^ and an elder abuse prevention and caregiver support law was enacted in 2006.^23^ Nevertheless, a new type of health inequality may result from imbalances and lack of harmonization in the community’s supply and demand of health and social care.^24^^,^^25^

Within the natural course of aging (eg, from independence in activities of daily living [ADL], with only minor comorbidities, to ADL-dependence due to stroke sequelae and/or other medical conditions), what do older people and their families want from society? Which health services are efficient and fair? To answer these questions, we urgently need evidence-based approaches to living arrangements, social support, and social capital that are based on social epidemiology, gerontology, and health services research.

The Aichi Gerontological Evaluation Study (AGES) project was launched for this purpose in 1999. At the Center for Well-being and Society of Nihon Fukushi University in Aichi prefecture, Japan, one of the authors (KK) and colleagues are responsible for managing the project and thus take full responsibility for it. The organizations that provided funding for this research had no role in the conduct of the study or the presentation of its results.

## METHODS

### Study design, setting, and participants

After a pilot cohort was generated and evaluated in 1999, we organized the AGES 2003 Cohort Study as a part of the AGES project. In this prospective study, the source population was community-dwelling individuals aged 65 years or older who lived in 6 targeted municipalities and were ADL-independent as of the second half of 2003 (the exact date varied by municipality). The targeted municipalities covered the entire southern part of the Chita peninsula in Aichi Prefecture (ie, Agui town, Handa city, Tokoname city, Taketoyo town, Mihama town, and Minami-Chita town). The 6 municipalities consist of 18 *kyuuson* (the second smallest administrative unit in Japan, based on the municipalities in 1950), which comprise urban, semi-urban, and rural settings.^9^ Older people who were not ADL-independent were excluded if they were eligible to receive benefits from public long-term care insurance (LTCI) services and or if they indicated that they were ADL-dependent in the baseline questionnaire.

The survey was conducted using a random sampling method in the 2 larger municipalities (Handa and Tokoname) and a complete census (complete enumeration) of the 4 smaller municipalities (Agui, Mihama, Minami-Chita, and Taketoyo) by municipal officers of the public LTCI system. A total of 49 707 residents aged 65 or older as of 1 October 2003 were targeted (Figure). The self-administered baseline questionnaire was mailed to 29 374 individuals selected by the sampling process as stated above. Then, 13 310 individuals (6508 men; 6802 women) were introduced to the AGES 2003 Cohort. There are no monetary or other incentives to encourage participation in the cohort. For data analysis, the municipality-level sample weight was calculated for selection probability, nonresponse, and other adjustments, to reflect the population proportion of those aged 65 or older in each municipality.

**Figure.**
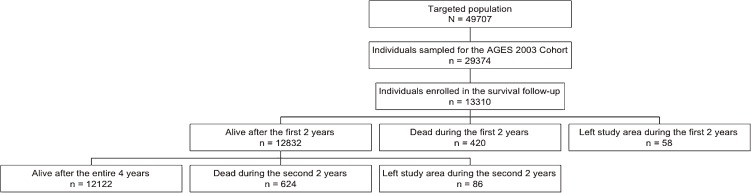
Flowchart of the AGES 2003 Cohort study in Japan

### Baseline measures

Table 1 summarizes the main measures that were collected in 2003. Other variables were also experimentally measured (some of which are not shown in Table 1). A summary of the measures is available at our website (http://square.umin.ac.jp/ages/index.html). The details of the questionnaire (including the exact wording of every question in Japanese and the translated version in English) have been published elsewhere.^26^^–^^28^

**Table 1.** Summary of data collected in the AGES cohort study

**Demographic factors**
**Individual-level living arrangements**
Cohabitation status and family structure
Marital status and marriage satisfaction
Caregiving arrangement (care of ageing relatives)
**Individual-level socioeconomic status**
Education
Household income
Work (retirement)
**Individual-level social support**
Emotional social support
Instrumental social support
**Individual-level social capital (participation)**
Vertical social capital
Horizontal social capital
**Psychological factors/exposures**
Sense of cohesion
Abuse/neglect
Negative life events
**Health-related and other behaviors**
Smoking
Alcohol consumption
Diet
Physical activity
Medical check-up
Hobby
**Community-level measures (6 municipalities with 18 *kyuuson*)**
Social capital (trust)
Other aggregated variables (GINI coefficient, etc)
Environmental factors ​ (Concentrations of SO_2_, NO_2_, CO, O_3_, PM, can be merged)
Address (geocoding)
**General and mental health (subjective)**
Self-rated health
Self-reported medical conditions
Medication
Sleeping
Activities of Daily Living (ADL)
Instrumental ADL
Geriatric Depression Scale (GDS)
Height, Weight, and Body Mass Index (BMI)
Dental status
**Health outcomes (objective)**
All-cause mortality (Survival time)
Eligible care-level in national long-term care insurance system

The baseline questionnaire included the following individual-level information: demographic factors (date of birth and sex), living arrangements (cohabitation status, marital status, and caregiving status), and socioeconomic status (education, household income, and current occupation status). Details of individual-level social support (receiving and providing emotional support and instrumental support), social participation, and sense of coherence^29^^,^^30^ were also obtained. Other factors included abuse of older people, negative life events (retirement, death of spouse, death of close relatives/friends, serious disease, a change in living arrangements such as moving, economic distress, or the beginning of informal family caregiving in the recent year), health-related behaviors (smoking, alcohol consumption, diet habits, physical activity [walking]), frequency of medical check-ups, and any hobbies. We regarded social participation as individual-level social capital and divided it into vertical and horizontal social capital. Vertical social capital was defined as participating in groups that encouraged hierarchical relations, and horizontal social capital was defined as participating in groups of social equals.^18^

Community-level measures were also obtained. Social capital (trust, reciprocity, and crime) was aggregated by individual-level variables.^31^ Other community-level variables of social factors (eg, the Gini coefficient) were aggregated by individual responses. For environmental exposures, information on airborne particle concentration (sulfur dioxide [SO_2_], nitrous monoxide [NO], carbon monoxide [CO], ozone [O_3_], and particulate matter with an aerodynamic diameter <10 µm [PM_10_])^32^ in the 6 municipalities (7 measuring points) was available from the Aichi prefecture website.^33^ We geocoded study participants’ home locations in each municipality, which enabled us to introduce municipality- or *kyuuson*-level multilevel analysis using Geographic Information System (GIS)-based methods.

Subjective general and mental health information included self-rated health,^34^ self-reported medical conditions (cancer, heart disease, stroke, hypertension, diabetes mellitus, hyperlipidemia, osteoporosis, joint/neurological disease, trauma/fracture, respiratory disease, gastrointestinal disease, liver disease, visual impairment, hearing impairment, urinary disorder, sleep disorder, and others), medication, sleeping, activities of daily living (Tokyo Metropolitan Institute of Gerontology Index of Competence [TMIG-IC]),^35^ Instrumental ADL,^36^ depression (Geriatric Depression Scale [GDS-15]),^37^^,^^38^ height, weight, body mass index (BMI), and dental status (number of remaining teeth).

### Follow-up and outcome measures

Survival time was monitored since the second half of 2003 (the time when self-administered questionnaires were distributed varied by municipality). When individuals died or moved during follow-up, the date they died or left the study area was recorded. We obtained information on physical and cognitive disability by using the LTCI database maintained by the municipalities. The qualification was based on a standardized multistep assessment with several levels of care need, ranging from support levels 1 and 2 to care levels 1 through 5.^21^

### Ethical issues

Our study protocol and informed consent procedure were approved by the Ethics Committee on the Research of Human Subjects at Nihon Fukushi University.

### Statistical analysis

All analyses were performed using the computer software STATA/IC 11.0 (StataCorp LP, College Station, TX, USA).

## RESULTS

The baseline characteristics of the participants are shown in Table 2. Living alone, being divorced/widowed, lower education status, no current occupation, no smoking, and no alcohol consumption were more frequent in women than in men. As age increased, the proportions of those with a tertiary education decreased, as did current smokers, drinkers, and positive horizontal social capital.

**Table 2.** Baseline characteristics of the AGES 2003 Cohort Study

	All	Men	Women	Age group (years of age)				
	
				65–69	70–74	75–79	80–84	85–
Number of individuals^a^	13 310	6508	6802	4685	3934	2819	1272	585

Birth year (age group)								
–1918 (85–)	4.3%	3.8%	5.7%	—	—	—	—	100.0%
1919–1923 (80–84)	9.5%	8.6%	11.2%	—	—	—	100.0%	—
1924–1928 (75–79)	21.5%	20.6%	23.1%	—	—	100.0%	—	—
1929–1933 (70–74)	30.1%	30.9%	28.7%	—	100.0%	—	—	—
1934–1938 (65–69)	34.5%	36.1%	31.4%	100.0%	—	—	—	—
Sex								
Men	49.9%	100.0%	—	52.3%	50.7%	46.0%	42.3%	38.5%
Women	50.1%	—	100.0%	47.7%	49.3%	54.0%	57.7%	61.5%
Cohabitation status								
No	10.4%	4.4%	16.3%	7.5%	10.9%	12.1%	13.6%	14.2%
Yes	89.6%	95.6%	83.7%	92.5%	89.1%	87.9%	86.4%	85.8%
Marital status								
Married	71.7%	88.6%	54.9%	83.2%	75.3%	65.0%	50.6%	31.2%
Divorced/Widowed	25.8%	10.2%	41.3%	14.5%	21.9%	32.5%	46.5%	67.5%
Unmarried/Other	2.5%	1.2%	3.9%	2.3%	2.8%	2.5%	3.0%	1.3%
Education								
Primary or lower secondary	59.2%	55.6%	62.8%	55.3%	59.6%	58.8%	67.2%	72.7%
Higher secondary	30.7%	29.8%	31.5%	32.1%	30.6%	32.4%	26.5%	19.2%
Tertiary education	10.2%	14.6%	5.7%	12.6%	9.8%	8.8%	6.3%	8.2%
Household income								
Bottom quartile	25.8%	21.7%	30.9%	23.1%	25.9%	28.0%	29.7%	32.1%
2nd quartile	21.4%	23.5%	18.8%	24.0%	21.4%	17.7%	20.3%	17.4%
3rd quartile	30.3%	32.3%	27.8%	31.2%	30.9%	30.7%	25.3%	24.3%
Top quartile	22.5%	22.5%	22.6%	21.7%	21.9%	23.6%	24.7%	26.2%
Occupation status								
No	75.2%	68.6%	81.8%	64.0%	74.8%	84.6%	88.9%	91.8%
Yes	24.8%	31.4%	18.2%	36.0%	25.3%	15.4%	11.1%	8.2%
Emotional social support								
Not receiving	10.8%	14.8%	6.8%	9.5%	11.5%	10.2%	13.9%	13.2%
Receiving	89.2%	85.2%	93.2%	90.5%	88.5%	89.8%	86.1%	86.8%
Instrumental social support								
Not receiving	6.3%	4.6%	8.0%	6.1%	6.8%	6.5%	5.5%	4.9%
Receiving	93.7%	95.5%	92.0%	93.9%	93.2%	93.5%	94.5%	95.1%
Vertical social capital								
No	40.7%	39.2%	42.3%	44.1%	39.3%	36.8%	38.5%	47.2%
Yes	59.3%	60.9%	57.8%	55.9%	60.7%	63.2%	61.5%	52.9%
Horizontal social capital								
No	61.2%	61.6%	60.7%	54.6%	59.1%	65.2%	72.7%	82.3%
Yes	38.8%	38.4%	39.3%	45.4%	40.9%	34.8%	27.3%	17.7%
Smoking								
Nonsmoker	60.0%	27.3%	93.0%	58.6%	58.0%	59.9%	66.1%	73.0%
Ex-smoker	26.4%	48.4%	4.3%	25.5%	27.7%	28.5%	24.6%	19.1%
Smoker	13.5%	24.3%	2.7%	15.9%	14.3%	11.6%	9.3%	7.9%
Alcohol consumption								
No/Abstinent	65.4%	43.6%	87.2%	57.5%	64.1%	71.6%	77.1%	82.6%
Occasional/Light	13.5%	18.1%	8.9%	17.4%	13.5%	10.0%	9.7%	7.8%
Moderate	16.4%	29.2%	3.6%	18.0%	17.4%	15.9%	11.8%	8.7%
Heavy	4.7%	9.1%	0.3%	7.2%	5.0%	2.5%	1.5%	1.0%
Physical activity								
≤30 minutes/day	36.6%	35.6%	37.7%	32.1%	38.6%	39.0%	40.2%	39.7%
>30 minutes/day	63.4%	64.4%	62.3%	67.9%	61.4%	61.0%	59.8%	60.3%
No. of self-reported diseases								
0	19.1%	19.8%	18.4%	25.6%	17.9%	14.2%	12.0%	15.0%
1–2	58.8%	59.3%	58.2%	57.5%	60.9%	58.2%	59.0%	55.7%
3–4	17.9%	17.4%	18.5%	14.5%	17.6%	21.2%	22.2%	22.9%
5–	4.2%	3.5%	5.0%	2.4%	3.6%	6.5%	6.8%	6.4%
Self-rated health								
Excellent	8.1%	8.8%	7.4%	10.8%	7.2%	5.8%	6.1%	8.2%
Good	65.3%	64.9%	65.7%	67.5%	66.8%	60.7%	63.0%	63.8%
Fair	22.6%	22.0%	23.1%	18.3%	21.9%	28.6%	26.4%	23.6%
Poor	4.1%	4.3%	3.9%	3.5%	4.0%	4.9%	4.5%	4.5%
GDS15								
0–4	71.3%	72.4%	70.1%	74.7%	70.4%	68.8%	68.4%	65.5%
5–9	23.6%	22.6%	24.8%	20.7%	23.6%	26.6%	27.5%	27.1%
10–	5.1%	5.0%	5.2%	4.6%	6.1%	4.6%	4.1%	7.5%

^a^Unweighted values are reported only for cells indicating numbers of individuals.

During a 48-month follow-up period, which is the current maximum, men were observed for 24 753 person-years and women for 26 456 person-years. In total, 0.8% of the study participants were lost to follow-up because they left the study area during the 4-year period (*n* = 105). The cumulative survival rate for the study population was 96.8% (men, 95.6%; women, 98.0%) at 24 months and 92.1% (men, 89.4%; women, 94.7%) at 48 months. Sex and age-group differences among individual-level core variables are shown in Table 2. Overall and sex-stratified cumulative survival rates at 24 and 48 months for individual-level core variables are shown in Table 3.

**Table 3.** Cumulative survival rates for different individual-level core variables in the AGES 2003 Cohort Study (*n* = 13 310)

	All		Men		Women	
			
	24 months	48 months	24 months	48 months	24 months	48 months
Birth year						
–1918	87.5%	69.6%	82.6%	62.0%	90.5%	74.3%
1919–1923	94.2%	85.9%	90.6%	78.7%	96.7%	91.1%
1924–1928	96.1%	90.1%	94.1%	85.2%	97.8%	94.2%
1929–1933	97.7%	94.3%	96.9%	92.1%	98.5%	96.6%
1934–1938	98.5%	96.0%	97.5%	94.2%	99.5%	97.9%
Sex						
Men	95.6%	89.4%				
Women	98.0%	94.7%				
No. of self-reported medical conditions						
0	98.0%	94.7%	97.6%	93.4%	98.5%	96.0%
1–2	97.0%	92.5%	95.8%	89.8%	98.2%	95.0%
3–4	95.4%	89.5%	93.4%	85.0%	97.1%	93.6%
5–	95.2%	86.3%	91.7%	79.1%	97.5%	91.0%
Marital status						
Married	97.0%	92.9%	96.0%	90.5%	98.6%	96.5%
Divorced/Widowed	96.5%	90.6%	92.9%	81.7%	97.3%	92.6%
Unmarried/Other	96.5%	91.8%	95.5%	89.5%	96.8%	92.6%
Smoking						
Nonsmoker	97.8%	94.2%	96.4%	91.1%	98.2%	95.1%
Ex-smoker	95.7%	90.4%	95.6%	90.2%	96.7%	93.3%
Smoker	95.0%	87.3%	94.9%	87.2%	95.3%	88.9%
Alcohol consumption						
No/Abstinent	96.5%	91.9%	93.5%	86.1%	97.9%	94.7%
Occasional/Light	97.6%	93.2%	97.1%	92.2%	98.5%	95.4%
Moderate	97.3%	92.3%	97.1%	91.8%	98.7%	96.1%
Heavy	98.0%	92.7%	97.9%	92.7%	100.0%	95.2%
Physical activity						
≤30 minutes/day	95.9%	89.7%	94.1%	85.4%	97.7%	93.8%
>30 minutes/day	97.3%	93.5%	96.5%	91.8%	98.2%	95.3%
Education						
Primary or lower secondary	96.6%	91.5%	95.2%	88.2%	97.9%	94.4%
Higher secondary	97.4%	93.3%	96.2%	90.9%	98.5%	95.4%
Tertiary education	96.6%	92.9%	96.3%	91.7%	97.4%	96.2%
Household income						
Bottom quartile	96.1%	90.6%	94.3%	86.5%	97.7%	94.0%
2nd quartile	96.8%	93.2%	95.7%	91.2%	98.4%	96.2%
3rd quartile	97.0%	92.2%	95.8%	89.4%	98.7%	95.9%
Top quartile	97.2%	93.2%	97.2%	92.4%	97.2%	94.2%
Emotional social support						
Not receiving	96.1%	89.5%	95.3%	87.7%	97.9%	93.1%
Receiving	97.0%	92.6%	95.8%	90.0%	98.1%	95.0%
Instrumental social support						
Not receiving	97.7%	93.5%	96.1%	89.4%	98.6%	95.6%
Receiving	96.8%	92.2%	95.6%	89.5%	98.0%	94.8%
Vertical social capital						
No	96.6%	90.9%	95.1%	87.5%	98.0%	93.9%
Yes	97.0%	92.9%	95.9%	90.5%	98.1%	95.4%
Horizontal social capital						
No	96.2%	90.4%	94.8%	87.3%	97.5%	93.4%
Yes	98.0%	94.9%	96.9%	92.8%	99.0%	96.9%

## DISCUSSION

The main strengths of the AGES 2003 Cohort Study are: (1) the location of the study—Japan leads the world in the pace of population aging, (2) the linkage with geographical data via the Geographic Information System, (3) the quality and length of follow-up—there is no administrative loss during follow-up, (4) the large variability of health determinants (health-related behavior, socioeconomic status, social support, and social capital at the individual and community level) minimizes omitted variable bias, and (5) the availability of multilevel analysis.

The main weaknesses of the study are the moderate response rate and the limited generalizability of the findings, as the data are not obtained from a national representative sample. Nevertheless, urban, semi-urban, and rural municipalities were included. Regarding the moderate response rate of the AGES 2003 Cohort Study, differences between respondents and nonrespondents at baseline were examined with respect to some demographic characteristics.^26^^,^^28^ Although the available information did not encompass all 6 municipalities, individuals who were younger than 80 years and those with a household income higher than the median were more likely to answer the baseline questionnaire (*P* < 0.10 and *P* < 0.001, respectively). There was no difference between men and women.

Our study group is currently conducting quantitative research using data on all-cause mortality from the AGES 2003 Cohort Study. However, several peer-reviewed articles and a book (and its English translation) have already been published on the AGES project.^9^^,^^18^^–^^20^^,^^26^^,^^28^ Using cross-sectional data from 2003, with a baseline that extended across 15 municipalities in 3 prefectures, Kondo and colleagues described the relationship between socioeconomic status and self-reported health status.^26^^,^^28^

Murata and colleagues reported that lower SES and residential area were significantly associated with depression.^20^ A study by Ichida and colleagues was the first in Japan to use multilevel analysis to support the relative income hypothesis.^9^ Aida and colleagues also used multilevel analysis to reveal that horizontal social capital but not vertical social capital had beneficial effects on the number of remaining teeth in older Japanese adults.^18^ Another study identified the risk factors for eligible care level under the national long-term care insurance from 2003 to 2006–2007. Kondo and colleagues reported that relative deprivation may be a mechanism underlying the relationship between income inequality and disability during old age, at least among men.

In conclusion, the AGES 2003 Cohort Study has provided useful and observable quantitative findings for use in social epidemiology, gerontology, and health services research. We reported the baseline profile and the progress of the AGES 2003 Cohort Study under the AGES project, which is an open, general-purpose epidemiological/gerontological laboratory. An additional survey was implemented in 2006–2007 and is planned for 2010. We are certain to obtain additional useful data in the 2010 survey. The data in the AGES project is available to academic investigators on an approval basis. The first step is to contact Katsunori Kondo. Please refer to the AGES project website (http://square.umin.ac.jp/ages/index.html) for details.^27^
